# Key community eye health messages

**Published:** 2017-05-12

## Managing your own information and resources is an important element of keeping up to date

**Figure:**
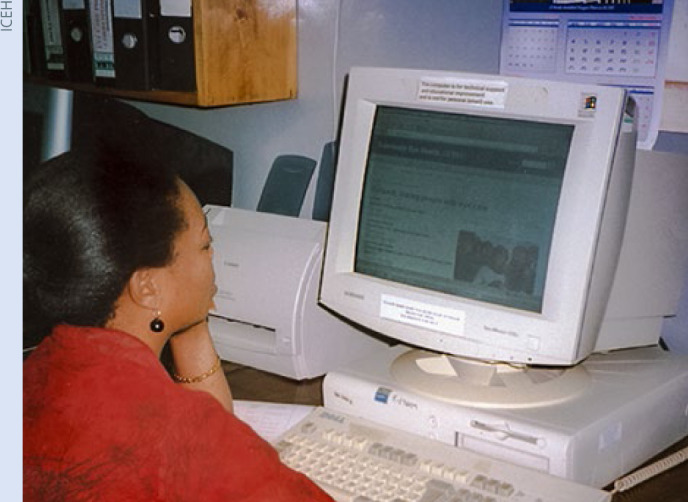

- Keep a paper diary or use an app
- Compile your own library of useful articles or web links
- Write down useful advice or tips from others
- Use digital alerts to stay up to date
- Keep all those CPD certificates!

## Continuing to learn is good for our patients and eye health professionals. CPD:

**Figure:**
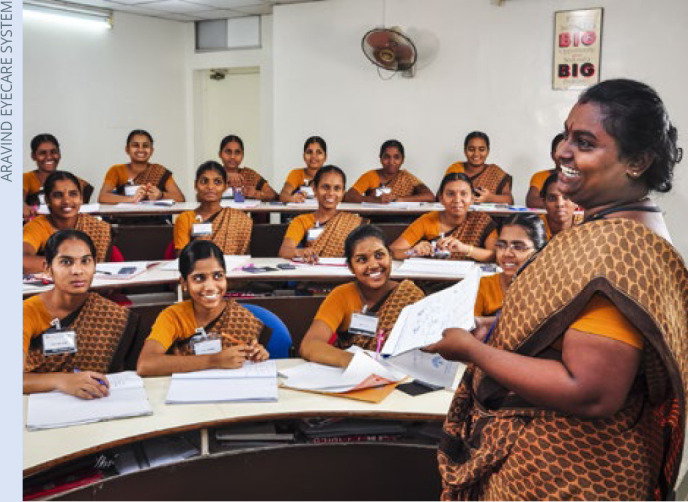

- Produces positive change in the participant's practice
- Instils confidence and self-esteem
- Facilitates the efficiency, effectiveness and quality of the eye care team
- Assures the best outcomes for the maximum number of patients
- Improves access to high quality eye care globally

## Professional organisations have a responsibility to support and implement CPD by being aware that:

**Figure:**
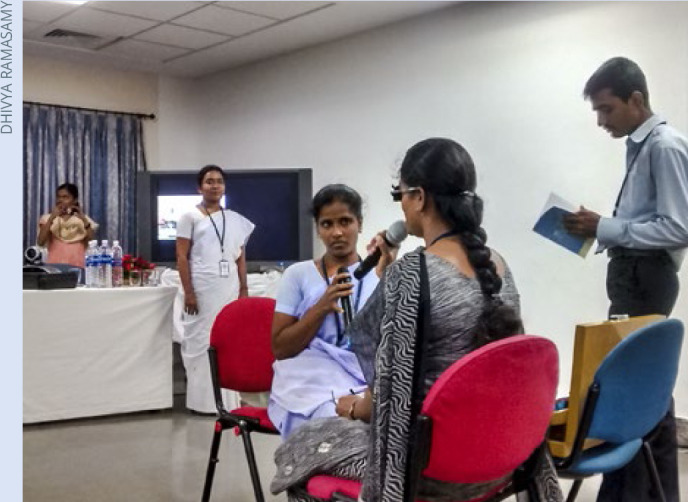

- CPD is a priority and directly leads to improved patient care
- All the relevant staff should be involved in CPD activities
- Learning outcomes should be defined at the start
- Barriers to learning must be overcome e.g. introducing practical demonstrations or role play
- Good communication with staff should be a priority

